# Behavioral Responses of the Bed Bug to Permethrin-Impregnated *Active*Guard™ Fabric

**DOI:** 10.3390/insects4020230

**Published:** 2013-06-07

**Authors:** Susan C. Jones, Joshua L. Bryant, Scott A. Harrison

**Affiliations:** Department of Entomology, Ohio State University, 2501 Carmack Rd., Columbus, OH 43210, USA; E-Mails: bryant.1310@osu.edu (J.L.B.); Harrison.308@osu.edu (S.A.H.)

**Keywords:** *Cimex lectularius*, behavior, feeding inhibition, insecticide-treated fabric, repellency

## Abstract

*Active*Guard™ Mattress Liners have been used to control house dust mites, and they also are commercially available as an integrated pest management tool for use against bed bugs (*Cimex lectularius*). The aim of our study was to evaluate responses of numerous populations of the bed bug to the permethrin-impregnated fabric, with particular regard to contact toxicity, repellency, and feeding inhibition. Continuous exposure to *Active*Guard fabric resulted in rapid intoxication for three of four populations, with 87 to 100% of moderately pyrethroid-resistant and susceptible bed bugs succumbing by 1 d. In comparison, a highly resistant population reached 22% mortality at 10 d. Video data revealed that bed bugs readily traversed *Active*Guard fabric and spent a considerable amount of time moving about and resting on it during a 12-h period. *Active*Guard fabric was non-repellent to bed bugs from five tested populations. Furthermore, significantly fewer bed bugs successfully fed to repletion through *Active*Guard fabric than through blank fabric for the five populations. With just 30 min of feeding exposure, mortality ranged from 4% to 83%, depending upon the bed bug strain. These laboratory studies indicate that *Active*Guard liners adversely affected bed bugs from diverse populations.

## 1. Introduction

Insecticide efficacy can be greatly influenced by the behavioral response of the target pest. Contact repellents, sometime referred to as contact irritants, require the insect to physically contact a chemical residue before making oriented movement away; these chemicals typically have low vapor pressure. In contrast, with vapor (olfactory, spatial) repellents, the insect orients its movement away from the chemical residue without having physically contacted it; such chemicals typically have a high vapor pressure [1,2,3]. 

There are limited data on repellency of insecticides to bed bugs, *Cimex lectularius* (Hemiptera: Cimicidae) [4,5]. Moore and Miller [4] provided the first insights into the non-repellency of formulated insecticides for bed bugs. They observed that bugs readily rested on panels treated with various pyrethroids: permethrin (Dragnet SFR), lambda-cyhalothrin (Demand CS), deltamethrin (Suspend SC), and bifenthrin (Talstar One), as well as a chlorinated pyrrole (chlorfenapyr [Phantom]). Hence, these researchers found that a number of currently registered insecticides generally exhibited little, if any, repellency toward bed bugs.

In contrast, in choice bioassays, Romero *et al.* [5] found that bed bugs spent significantly more time resting on untreated tent harborages than on deltamethrin-treated harborages, but the bugs readily rested on chlorfenapyr-treated harborages. In no-choice bioassays, bed bugs spent a greater proportion of time away from tent harborages that had been treated with sublethal rates of deltamethrin compared to control tents; this was not observed for chlorfenapyr. However, in follow-up bioassays using harborages that were conditioned with bed bug feces and unspecified pheromones, deltamethrin was no longer repellent to the bugs. Furthermore, bed bugs readily crossed surfaces treated with deltamethrin or chlorfenapyr to obtain a blood meal. Hence, there is some indication that bed bugs may avoid pyrethroid-treated surfaces in the absence of aggregation cues or host attractants. 

*Active*Guard™ Mattress Liners (Allergy Technologies LLC, Ambler, PA, USA) are comprised of finely woven polyester fabric that is impregnated with permethrin (1.64%) through a non-covalent bonding process that allows for bioavailability of permethrin sufficient to kill select arthropods including house dust mites (*Dermatophagoides* spp.) and bed bugs. The liners are fitted onto the mattress and box springs similar to a fitted sheet, then kept in place beneath mattress pads, sheets, or similar bedding. The liners do not serve as a physical barrier to arthropods, but rather, they are intended to kill those arthropods that maintain contact with the bioavailable permethrin on the surface of the liners. The product has residual activity for up to 2 y.

*Active*Guard liners originally were developed to reduce asthma-associated allergens from house dust mites [6,7]. More recently, with the worldwide resurgence of bed bugs, *Active*Guard is recommended as the last step in an integrated pest management (IPM) program to remediate an active bed bug infestation or to prevent future infestation. For example, in a 40-room transitional housing facility, Ballard *et al.* [8] demonstrated that the number of bed bug infestations was reduced from an initial 45% bed bug infestation rate to just occasional re-introductions from new residents when using a multi-faceted approach that included *Active*Guard liners. Clutter reduction was the first step, followed by insecticide treatment of all rooms using Steri-Fab (isopropyl alcohol and phenothrin) for mattresses, Tempo 1% dust (cyfluthrin) for voids, and Transport GHP (acetamiprid and bifenthrin) for non-sleeping surfaces. Once rooms were cleared of bed bugs, either *Active*Guard liners or an encasement was installed on all mattresses. Monthly canine inspections detected bed bugs in several rooms during the first 2 mo after treatment; for the next 10 mo, an occasional room was found with bed bugs after being occupied by a new resident. However, live bed bugs were found only on beds with an encasement, not on beds with *Active*Guard liners.

The issue of potential repellency is important for an insecticide-impregnated liner because bed bugs must remain in contact with the fabric in order to obtain a toxic dose. The objectives of our study were threefold: (1) to determine the physical condition of bed bugs in continuous contact with *Active*Guard fabric, (2) to determine if *Active*Guard fabric was repellent to bed bugs, and (3) to determine how bed bugs would respond if they fed through *Active*Guard fabric. 

## 2. Methods

### 2.1. Bed Bug Populations

Five bed bug populations were tested in this study. Three of these were field populations (EPM and Marcia were collected from Columbus, OH, during August 2010; Earl was collected from Modesto, CA, USA, in 2007 [acquired from Sierra Research Laboratory in 2012]). Two were long-term laboratory strains (Harlan was collected in 1973 from Ft. Dix, NJ; FV was collected in 1987 from Dublin, OH, USA).

Pyrethroid resistance was characterized for bed bugs on the basis of deltamethrin bioassays, with most populations also evaluated for knockdown resistance (kdr) mutations [9]. For the bioassays, groups of 10 adult bed bugs from each population were exposed for 24 h to dry residues of deltamethrin (technical grade, 99% purity; Chem Service, West Chester, PA, USA) at five concentrations ranging from 0.0005 to 5 mg/cm^2^ as well as an acetone control. Results indicated that Marcia was highly pyrethroid-resistant (no mortality at the highest rate), EPM and FV were moderately pyrethroid-resistant, and Earl and Harlan were pyrethroid-susceptible.

DNA analysis indicated that both Marcia and EPM possessed two kdr mutations (L925I and V419L) in the voltage-gated sodium ion channels, whereas FV had only the V419L mutation. The Harlan strain showed neither mutation. Kdr screening has not been performed for Earl. 

### 2.2. Bed Bug Rearing

In the laboratory, each bed bug population was housed in a glass jar (13 cm high by 7 cm dia; narrow-mouth Mason pint jar, Ball Corp., Broomfield, CO, USA) containing folded filter paper strips for harborage, with an organza fabric and filter paper covering held in place with rubber bands and a screw-on metal ring. All bed bug populations were maintained in an environmental chamber in the laboratory (29 °C, 50% RH, 12:12 L:D).

Bed bugs were reared *in situ* on a diet of warmed defibrinated rabbit blood using the Hemotek 5W1 system (Discovery Workshops, Accrington, UK) equipped with five FU1 feeders. Parafilm was used as the membrane to cover the individual meal reservoirs, each of which holds approximately 5 mL; the feeding area of membrane is approximately 10 sq cm. Bed bugs were offered a blood meal about every 7 d, with each feeding bout typically lasting 30 min.

### 2.3. Contact Bioassay

Each test arena consisted of a ventilated polystyrene petri dish lid (100 mm by 25 mm; Fisherbrand, Fisher Scientific, Pittsburgh, PA, USA) that was secured with rubber bands (rim facing downward) atop a plywood panel (15.2 cm by 15.2 cm) that had been completely covered with a square of fabric, either *Active*Guard fabric (treatment) or blank fabric (control). Fluon (INSECT-a-SLIP, BioQuip Corp., Rancho Dominguez, CA, USA) was applied to the inner walls of the dish to help confine bed bugs to the arena. Ten mixed-sex adult bed bugs were placed inside each arena for continuous contact with the fabric with ten replicates per population. Four bed bug populations were used in this experiment: Harlan, Earl, EPM, and Marcia.

The condition of bed bugs was assessed at 1 and 4 h, 1, 3, 6 and 10 d. Each bug’s condition was assessed based on its behavioral response when probed:

- Healthy—the bed bug moves quickly and in a coordinated manner to avoid stimulus.
- Sluggish—reacts slowly, but makes coordinated movements to avoid stimulus.
- Ataxic—unable to coordinate movements to avoid the stimulus. Ataxic bugs can right themselves after falling.
- Moribund—incapable of locomotion and exhibiting movement only of appendages or other body parts.
- Dead—no movement whatsoever.

### 2.4. Repellency Bioassay

Each test arena consisted of an open polystyrene petri dish (100 mm by 15 mm) with the bottom entirely lined with two conjoined semicircles of fabric affixed in place with spray adhesive (Craftbond, Elmer’s Products Inc., Westerville, OH, USA). (In preliminary tests, this spray adhesive did not cause bed bug mortality or affect performance of *Active*Guard fabric.) Fluon was applied to the inner walls of the dish.

Repellency was evaluated in choice test conditions. Treatment arenas were comprised of a semicircle of *Active*Guard fabric (treated zone = zone 1) and a semicircle of blank fabric (untreated zone = zone 2). Controls contained two semicircles of blank fabric, with the left and right side arbitrarily designated as zone 1 and zone 2, respectively.

An individual adult bed bug of known sex was introduced into the center of each treatment or control arena, and its movement was documented for 12 h (17:00 to 05:00) using a Sony HDR recorder. For videotaping purposes, 12 test arenas, configured in 3 rows and 4 columns, were positioned on a tray surrounded by a water moat to further impede potential escapees. Each bout of videotaping encompassed six treatments and six controls from the same population. Although 24 to 36 replicates of the treatment and control were established for each of the five bed bug populations, a bug occasionally escaped sometime during video acquisition which led to different sample sizes among populations. Nonetheless, total replicates ranged from 18 to 29 per population. During the breakdown of each trial, which took place ~18 h after setup, the condition of each bug was assessed.

Video was imported into EthoVision XT behavior research software (version 8.5, Noldus, Wageningen, The Netherlands) where movement of each bug was tracked (sample rate = 2 per s) for analysis. The following parameters were quantified for each bed bug: amount of time spent in each zone, number of times that a transition occurred between zones, total distance traveled, and velocity. 

### 2.5. Feeding Inhibition Bioassay

Each test arena consisted of a plastic cylindrical chamber (5.5 cm dia by 3.8 cm ht) whose aperture was covered with a piece of *Active*Guard fabric or untreated fabric (control), and containing 10 adult bed bugs. Each arena was positioned on its side abutting a parafilm-covered blood-filled meal reservoir. A 2.4 cm-wide strip of masking tape affixed lengthwise on the inner wall of each chamber provided bugs an easily navigable route to the fabric covering where bugs had to be positioned in order to feed. 

Bugs were starved for 14 d, then they were allowed 30 min to initiate feeding. (In preliminary tests, 30 min was sufficient for 100% of Harlan bugs to feed to repletion through a blank liner.) The number of bugs that fed to repletion (as evidenced by their visibly distended abdomen) was recorded. Five replicates were evaluated for each of the five bed bug populations.

After the 30 min feeding period, a filter paper disc (4.25 cm, Whatman no. 1) was added to each arena and any *Active*Guard fabric was removed and replaced with a swatch of blank fabric. The condition of each bug was assessed at 1, 2, 4, and 7 d based on its behavioral response when probed.

### 2.6. Data Analysis

Mortality data, which included moribund and dead bugs, was evaluated for the contact and feeding inhibition bioassays. These data were corrected for control mortality by applying Abbott’s formula [10]. However, when overall mean control mortality at an observation time exceeded 15%, all data for that observation were considered unreliable and were not analyzed. The corrected data were subjected to repeated-measures ANOVA using Statistica 6.1 (StatSoft Inc., Tulsa, OK, USA), with observation time as the repeated-measures factor and population and treatment as categorical predictor variables. For the feeding bioassay, the percentage of replete bed bugs was subjected to a two-way ANOVA with treatment and population as the factors. Tukey’s HSD test was used for *post hoc* comparison of means for significant main effects and interaction effects. In repellency bioassays, a *t*-test was used to compare differences between treatments and controls for each population. 

## 3. Results and Discussion

### 3.1. Contact Bioassay

**Figure 1:**
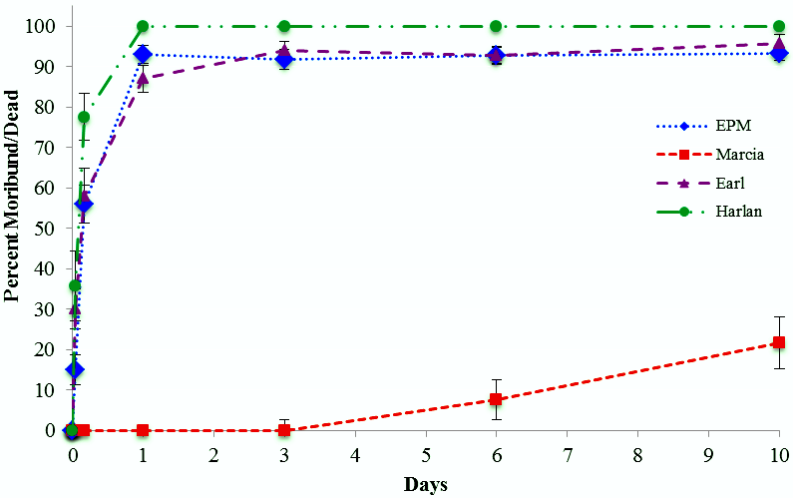
Cumulative percentage corrected mortality (mean ± SE) of bed bugs from four populations (*n =* 10 reps/population, with 10 bugs/rep) in continuous contact with *Active*Guard fabric.

Continuous contact with *Active*Guard fabric resulted in rapid intoxication of bed bugs from three of four tested populations, with 100% of Harlan, 93% of EPM, and 87% of Earl bed bugs succumbing by 1 d (Figure 1) and no significant differences evident by day 3 based on means separation tests. These analyses indicated that there was variability among populations (*F* = 395.4; df = 3, 36; *p <* 0.001) depending on observation time (F = 145.0; df = 5, 180; *p <* 0.001), with a significant interaction effect (F = 13.4; df = 15, 180; *p <* 0.001). The highly resistant population, Marcia, exhibited delayed mortality; some moribund and dead bed bugs were first observed at 6 d, with mortality subsequently increasing to 22% at 10 d (Figure 1). 

Based on these data, moderately pyrethroid-resistant bed bugs appear to be quite susceptible to permethrin as formulated in the *Active*Guard fabric. However, highly pyrethroid-resistant bed bugs may require prolonged contact with the *Active*Guard fabric to be killed. 

### 3.2. Repellency Bioassay

**Figure 2:**
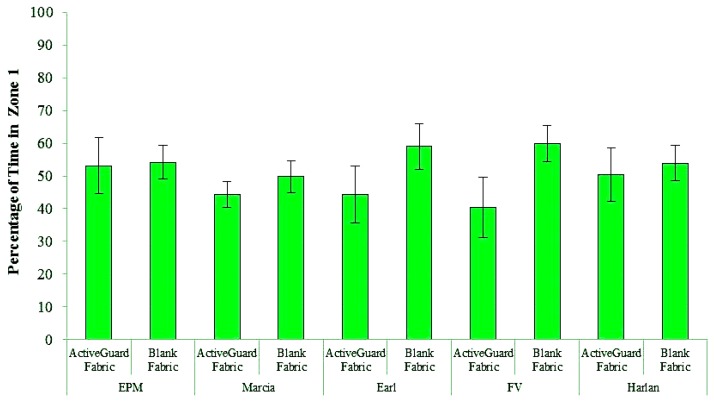
The percentage of time (mean ± SE) that bed bugs (5 populations) spent in zone 1 on the semicircle of *Active*Guard fabric in treatment arenas or on blank fabric in control arenas during a 12-h period in choice bioassays.

*Active*Guard fabric was non-repellent to bed bugs from all five tested populations (Figure 2, Table 1). There was no significant difference in the time that bed bugs spent on the semicircles of blank fabric and *Active*Guard fabric. In these choice bioassays, bed bugs readily traversed the *Active*Guard fabric and spent a considerable amount of time moving about and resting on it during a 12-h observation period.

**Table 1:** Mean and standard error for movement parameters associated with bed bugs (*N =* total number) that were videotaped for 12 h in a choice bioassay. For each population, means in the same column followed by the same letter are not significantly different (two-tailed *t*-test, α = 0.05).

Population	Treatment	*N*	Time Spent in Zone 1 ^1^ (s)	Distance Traveled (cm)	Velocity (cm/s)	Number of Zone Transitions	Percentage Affected ^2^
EPM	Treatment	24	22,959 a ± 3,675	732.4 a ± 113	0.017 a ± 0.003	23.4 a ± 4.1	70.8
	Control	22	23,419 a ± 2,215	3,627.8 b ± 648	0.084 b ± 0.015	142.1 b ± 27.2	4.5
Marcia	Treatment	19	19,147 a ± 1,737	7,481.3 a ± 837	0.191 a ± 0.021	307.6 a ± 35.4	0
	Control	21	21,492 a ± 2,095	7,156.6 a ± 856	0.171 a ± 0.019	301.0 a ± 39.3	0
Earl	Treatment	21	19,135 a ± 3,732	654.1 a ± 115	0.015 a ± 0.003	27.3 a ± 6.2	47.6
	Control	20	25,497 a ± 3,005	2,305.3 b ± 268	0.059 b ± 0.007	95.8 b ± 11.4	5
FV	Treatment	29	17,437 a ± 3,549	511.8 a ± 77	0.012 a ± 0.002	17.0 a ± 2.3	3.4
	Control	29	25,857 a ± 2,307	824.1 b ± 91	0.020 b ± 0.003	34.8 b ± 4.3	0
Harlan	Treatment	23	21,778 a ± 3,943	436.6 a ± 49	0.010 a ± 0.001	15.7 a ± 2.9	78.3
	Control	18	23,299 a ± 2,379	2,175.8 b ± 330	0.051 b ± 0.008	105.4 b ± 13.8	0

^1^ Treatment arenas consisted of a semicircle of *Active*Guard fabric (zone 1) abutting a semicircle of blank fabric (zone 2). Controls contained two semicircles of blank fabric, with the left and right side arbitrarily designated as zones 1 and 2, respectively; ^2^ Ataxic, moribund, and dead bed bugs after 18 h.

**Figure 3:**
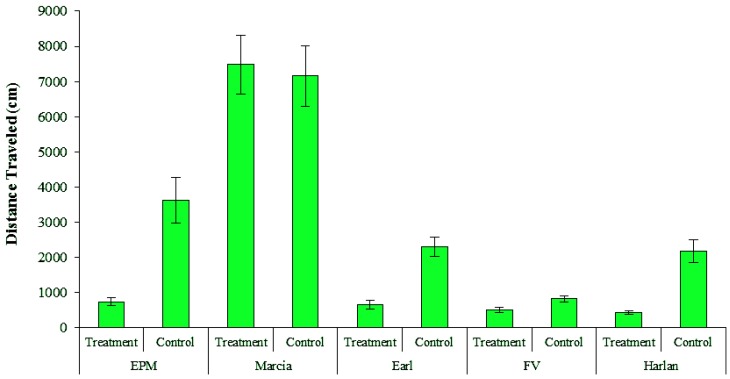
Total distance traveled (mean ± SE) during a 12-h period for bed bugs from five populations in choice bioassays with *Active*Guard fabric (treatment) and blank fabric (control).

Male and female bed bugs traveled similar distances for all populations. However, variability among bed bug populations was evident for total distance traversed during the 12-h test period (Figure 3); Marcia bed bugs, the highly resistant population, were more active than the other four populations. Marcia bugs traveled a greater distance than any other population, averaging 7,481.3 and 7,156.6 cm in treatment and control arenas, respectively (Table 1). These distances are not unexpected given that Suchy and Lewis [11] reported that male and female bed bugs moved at average speeds of 0.099 and 0.133 cm/s in the absence of host cues, respectively, and 0.384 and 0.438 cm/s when host breath (CO_2_) was present*.* These average velocities translate to distances ranging from approximately 5,010 to 17,751 cm during a 12-h period for bed bugs without and with host attractants, respectively. Hence, our data correspond with previous studies. However, further investigation is required to determine if bed bug activity is related to pyrethroid resistance levels.

Movement parameters such as distance traveled, velocity, and zone transition frequency were less in treatments than in controls for all populations except Marcia. It is likely that treatment effects resulted from permethrin intoxication from the bugs frequently contacting the *Active*Guard fabric. In several populations, many bugs in treatment arenas became intoxicated during the 12 h videotaping period whereas little, if any, mortality was evident in control arenas. When arenas were dismantled at ~18 h, Earl, EPM and Harlan bugs had relatively high levels of intoxication, with 47.6, 70.8 and 78.3% affected bugs, respectively; FV and Marcia had very few affected bugs with 3.4 and 0% affected bugs, respectively. One likely explanation for so few intoxicated FV bugs is that they were very inactive relative to the other populations (Figure 3). Their reduced movement on the *Active*Guard fabric potentially resulted in less accumulation of permethrin on the bugs’ cuticle resulting in fewer intoxicated bugs. It was expected from contact bioassays that Marcia, a highly resistant population, would not show immediate mortality effects.

### 3.3. Feeding Inhibition Bioassay

**Figure 4:**
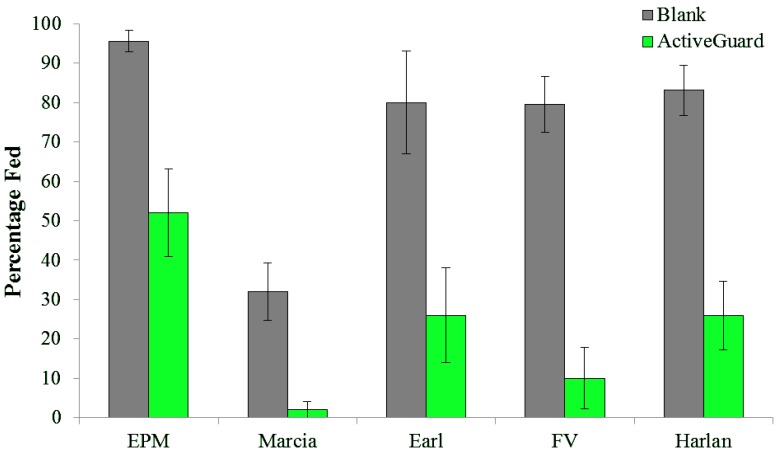
Percentage (mean ± SE) of bed bugs from five populations (*n =* 10 bugs; 5 reps) that fed to repletion during a 30-min period through blank fabric and *Active*Guard fabric.

Bed bugs exposed to *Active*Guard fabric exhibited significant feeding disruption compared to those on blank fabric (Figure 4). For all five bed bug populations, significantly fewer bed bugs successfully fed to repletion through *Active*Guard fabric than through blank fabric (F = 88.29; df = 1, 40; *p <* 0.001). The developmental and reproductive impact for bed bugs that did not feed to repletion is unknown.

Population was a significant factor (*F* = 11.62; df = 4, 40; *p <* 0.001) because some populations fed more than others; Marcia bugs fed the least (Figure 4). We frequently observe that bed bug populations differ in their feeding rates on artificial feeding systems. In our laboratory, Harlan, a long-term laboratory strain, readily feeds whereas recently field collected bugs often feed very poorly, necessitating numerous feeding bouts each week in order to sustain and grow populations. Chin-Heady *et al.* [12] reported extremely variable feeding rates for both a laboratory strain (University of Florida) and a field strain (Indianapolis, IN, USA) reared on rabbit blood using a water bath method and a petri dish system. Bed bug feeding in their study typically was less than 50%, and as low as 0% in some weeks and as high as 75% in others. In contrast, Montes *et al.* [13] reported 90 to 100% feeding for all bed bug stages using a water bath method; their population had been reared in the laboratory for >12 y.

Resultant bed bug mortality from brief feeding exposure (≤30 min) to *Active*Guard fabric varied among the populations (*F* = 16.93; df = 4, 20; *p <* 0.001). At 24 h, Marcia, FV, EPM, Earl, and Harlan exhibited 4, 17, 37, 50, and 83% corrected mortality, respectively. Mortality remained relatively close to these initial levels throughout the remainder of the 7-d observation period (*F* = 1.01; df = 3, 60; *p =* 0.393). In general, mortality paralleled feeding response, with higher mortality for populations that fed the most through the *Active*Guard fabric. EPM bugs were an exception as they had the highest feeding rate but intermediate mortality. Nonetheless, with just 30 min of feeding exposure, numerous bed bug strains exhibited high mortality.

### 3.4. Implications and Future Research

*Active*Guard has been successfully used as a continuous preventive/curative tool in bed bug treatment programs in facilities with high occupant turnover such as hotels and transient housing [6,8]. Data generated herein indicate a lack of repellency by the product that is consistent with *Active*Guard’s intended purpose to kill bed bugs that remain in contact with the bioavailable permethrin on the liner surface. Mortality of a variety of resistant populations is generally consistent with expectations given reported success with *Active*Guard in various locations throughout the U.S. [6,8] where one could expect to encounter resistant bed bug strains given their ubiquitous distribution nationwide [9].

The influence of permethrin on bed bugs through their exposure to the *Active*Guard fabric appears to be multifaceted. For susceptible and moderate to highly resistant bed bug strains, feeding inhibition was an early measureable behavioral response, often quickly followed by mortality. For susceptible bed bug strains, mortality is so rapid on *Active*Guard fabric that it may precede any feeding inhibition when the product is actually used on bedding. The effect of *Active*Guard on feeding inhibition of bed bugs appears to be independent of their resistance status. Permethrin may be an irritant, which would help explain its adverse effects on feeding success. Feeding inhibition is a novel finding that may help in the design, implementation, and interpretation of future experiments. 

Laboratory and field studies are planned to further investigate the activity of *Active*Guard against bed bugs. Research will be conducted to determine: permethrin formulation effects on resistant populations; methodology to enhance bioavailability of permethrin; and the impact of feeding inhibition on oviposition by females and on growth and development of nymphs. Standalone preventative activity of *Active*Guard will be investigated in a variety of room environments.

## 4. Conclusions

In a series of laboratory bioassays, *Active*Guard fabric adversely affected bed bugs from diverse populations. Continuous exposure to permethrin-impregnated *Active*Guard fabric resulted in rapid intoxication of several moderately pyrethroid-resistant and susceptible bed bug populations whereas a highly resistant population exhibited delayed mortality. In choice repellency tests, bed bugs from all five tested populations spent similar amounts of time on *Active*Guard fabric as on blank fabric, thereby demonstrating a lack of avoidance. Hence, *Active*Guard fabric was not repellent to bed bugs. Nonetheless, exposure to *Active*Guard fabric slowed the movement of bed bugs from some populations. In other bioassays, significantly fewer bed bugs successfully fed to repletion through *Active*Guard fabric compared to blank fabric; with just 30 min of feeding exposure, numerous bed bug strains exhibited high mortality.
